# Tracheostomal stenosis 10 years after total laryngectomy managed with bilateral advancement flap stomaplasty: a case report

**DOI:** 10.1093/jscr/rjag310

**Published:** 2026-04-30

**Authors:** Rosanna Xiang-Ying Tay, Nadia Hui Shan Sim, Jolie Hwee, Ming Yann Lim, Hui Wen Ng

**Affiliations:** Plastic, Reconstructive and Aesthetic Surgery Service, Department of General Surgery, Tan Tock Seng Hospital, Singapore, Singapore; Plastic, Reconstructive and Aesthetic Surgery Service, Department of General Surgery, Tan Tock Seng Hospital, Singapore, Singapore; Department of General Surgery, Khoo Teck Puat Hospital, Plastic, Reconstructive and Aesthetic Surgery Service, Singapore, Singapore; Department of Otorhinolaryngology, Tan Tock Seng Hospital, Singapore, Singapore; Plastic, Reconstructive and Aesthetic Surgery Service, Department of General Surgery, Tan Tock Seng Hospital, Singapore, Singapore

**Keywords:** stomaplasty, tracheostomal stenosis, laryngectomy, tracheostoma, stenosis

## Abstract

Tracheostomal stenosis is a recognized complication following total laryngectomy, with most cases occurring within the first post-operative year. We report a case of late-onset tracheostomal stenosis in a 67-year old male, presenting 10 years after total laryngectomy, bilateral neck dissection and adjuvant radiotherapy for laryngeal squamous cell carcinoma. The patient developed progressive breathlessness on neck flexion and examination revealed a stenotic tracheostoma measuring 7 × 10mm. The patient underwent stomaplasty using bilateral advancement flaps with lateral tracheal division and flap interposition, preserving the posterior tracheal wall for potential tracheoesophageal voice rehabilitation. At 2 months follow-up, the stoma remained patient at 18 × 20 mm, with resolution of symptoms. This report highlights the potential for delayed tracheostomal stenosis years after laryngectomy and describes a straightforward and effective method for its management.

## Introduction

Tracheostomal stenosis following total laryngectomy can compromise airway patency and speech rehabilitation. Patients may experience exertional dyspnea, difficulty clearing secretions, impaired voice prostheses use and reduced quality of life [1]. Severe stenosis may necessitate urgent intervention.

Most cases develop within the first post-operative year [2, 3]. We report a case of late onset tracheostomal stenosis presenting 10 years after total laryngectomy, managed with bilateral advancement flap stomaplasty.

## Case report

A 67-year-old male with a history of pT4aN0M0 laryngeal squamous cell carcinoma presented with progressive tracheostomal stenosis 10 years after total laryngectomy, bilateral neck dissection and adjuvant radiotherapy. The original tracheostoma had been constructed using a beveled technique. His postoperative course had been complicated by peristomal cellulitis that resolved with antibiotics. He used an electrolarynx for speech but was dissatisfied with speech quality. He remained well on annual follow-up until his tenth post-operative year, when he developed progressive breathlessness on neck flexion and in certain positions, beginning a few months prior to presentation.

**Figure 1:**
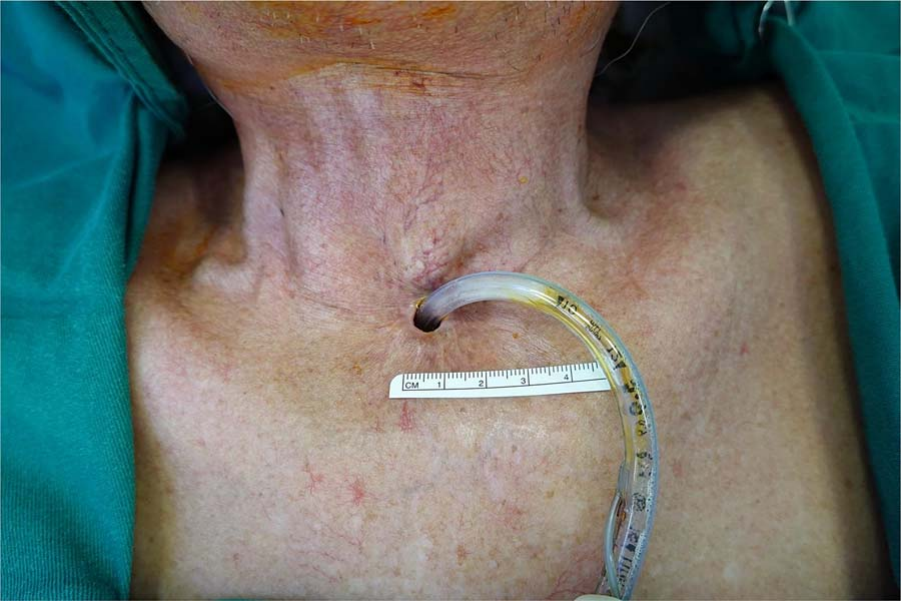
Pre-operative appearance of the tracheostoma measuring 7 × 11 mm, demonstrating concentric stenosis caused by overhanging skin and scar tissue.

Examination revealed a stenotic tracheostoma measuring 7 × 11 mm (Fig. 1) with no palpable neck masses. Flexible endoscopy confirmed a patent distal trachea and computed tomography showed no locoregional recurrence.

### Surgical technique

Stomaplasty was performed under general anaesthesia. Intra-operatively, the stenosis was found to be caused by a concentric ring of overhanging scar at the tracheocutaneous junction, consistent with radiation-induced fibrosis.

**Figure 2:**
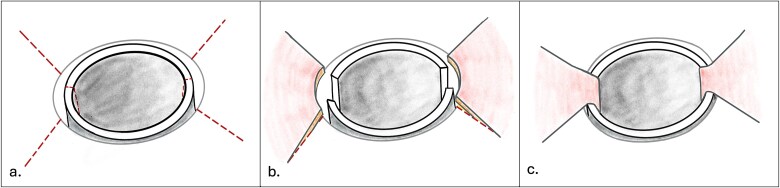
Illustration of surgical technique. (a) Radial incisions at the 2, 4, 8, and 10 o’clock of the positions of the stomal circumference and axial incisions on lateral tracheal walls. (b) Elevation of laterally based skin flaps. (c) Advancement of skin flaps and interposition into the lateral tracheal wall to widen the effective circumference of the stoma.

The procedure consisted of three main steps (Fig. 2).

#### Cicatricial excision and flap elevation

**Figure 3:**
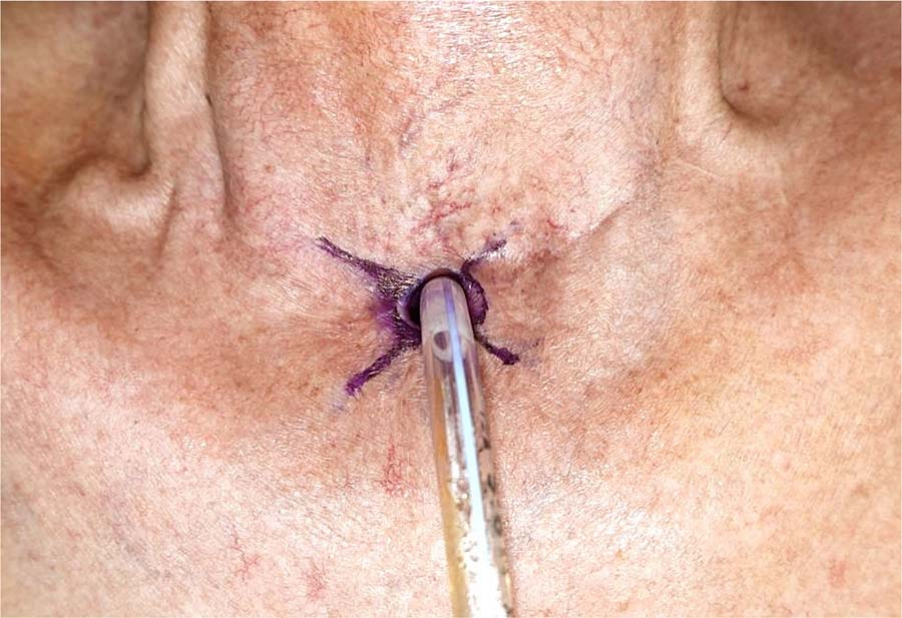
Surgical markings showing radial incisions at the 2, 4, 8 and 10 o’clock of the positions of the stomal circumference.

**Figure 4:**
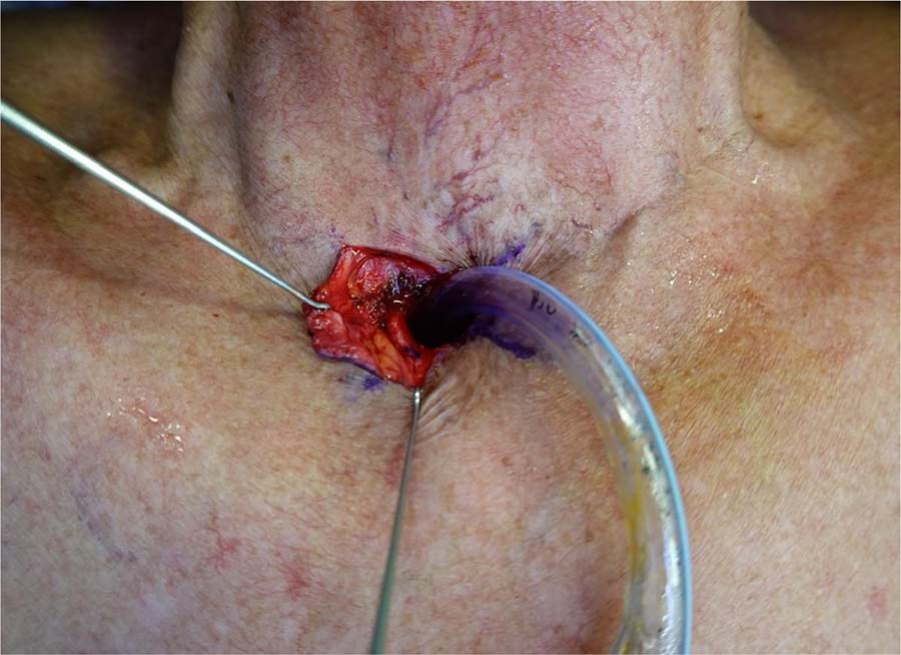
Elevation of laterally based skin flaps, demonstrating broad based advancement flaps.

Radial incisions were planned at the 2,4,8 and 10 O-clock positions (Figs 2a and 3). The overhanging scar was excised from the left and right sides of the stoma, sparing the posterior and anterior aspects. Laterally based skin flaps were elevated (Figs 2b and 4).

#### Lateral tracheal release and flap interposition

Additional scar along lateral tracheal walls was excised and the superior tracheal ring exposed. Dissection proceeded in the paratracheal plane, taking care to avoid the innominate vessels. The lateral tracheal walls were incised, creating wedge-shaped openings for flap interposition (Fig. 2b).

#### Closure

**Figure 5:**
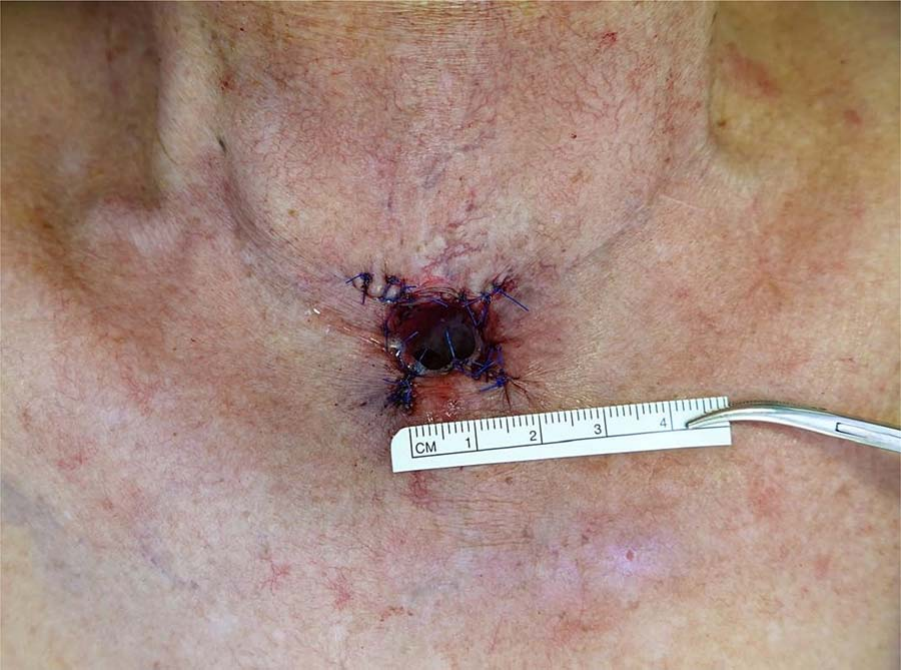
Following flap inset, the stoma measured 15 × 11 .

Skin flaps were advanced into the lateral tracheal wall incisions and inset using absorbable sutures (Fig. 2c). Immediate post-operative stoma size was 8 × 15 mm (Fig. 5). A size 6 Shiley tracheostomy tube was placed as a temporary stent and removed on post-operative Day 2.

**Figure 6:**
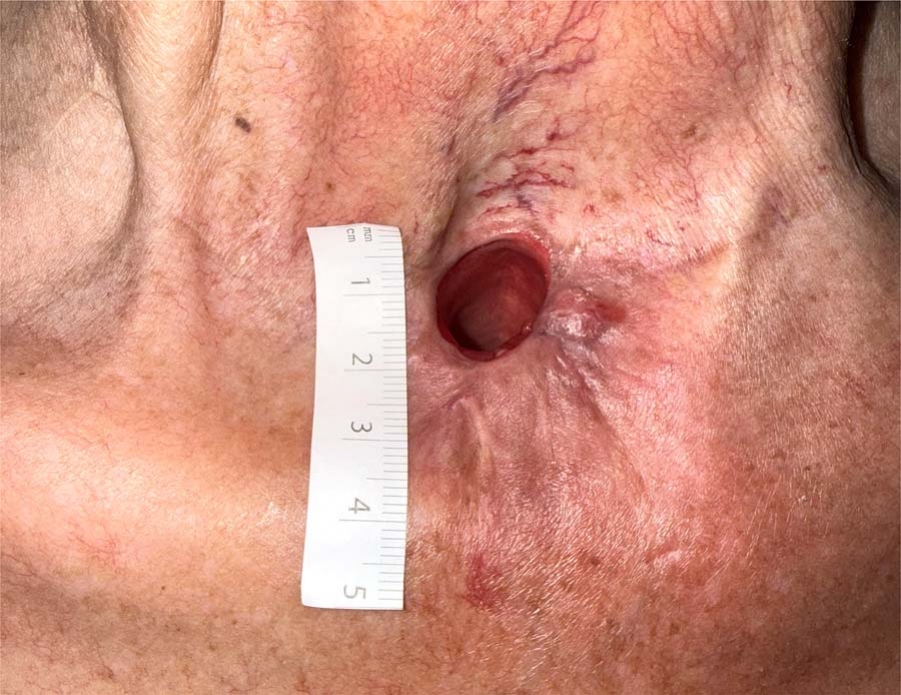
At post-operative 2 months, the stoma measured 18 × 20 mm.

Recovery was uneventful. At 2-month follow up the patient was asymptomatic, with a stoma measuring 18 × 20 mm (Fig. 6).

## Discussion

Tracheostomal stenosis occurs in 4% to 42% of patients following total laryngectomy [1]. Data regarding time to onset remains limited. In Lam’s series, all cases occurred within 11 months of laryngectomy [2]. de Virgilio’s series reported that 79% of cases developed within the first post-operative year, without specifying the timing of the remaining 21% [3] (Table 1). The minimum time of follow up in those studies was 9 and 18 months respectively. Seo described stenosis occurring 7 years after salvage laryngectomy [4] and Wax reported time to stomal revision ranging from 1–150 months, suggesting symptomatic stenosis may develop up to 12.5 years postoperatively [5].

**Table 1:** Time to onset of tracheostomal stenosis after total laryngectomy

Author, year	Number of patients	Onset	Follow up period	Prior radiotherapy, Number of patients(%)
Lam *et al.*, 1983 [2]	141	1–11 months	9 months – 16 years	57 (49%)
de Virgilio *et al.*, 2013 [3]	85	79% of cases onset within 1^st^ year	18 months minimum	85 (100%)
Seo *et al.*, 2022 [4]	1	7 years	Not reported	1 (100%)

Large cohort data on late-onset stenosis are constrained by short follow up periods, patient attrition from disease recurrence and mortality, as well as patients lost to follow up, likely underestimating the true incidence of delayed presentations.

This case is notable for its onset of tracheostomal stenosis 10 years after laryngectomy, and underscores the importance of long term follow-up. Progressive radiation-induced fibrosis, microvascular injury and chronic inflammatory changes may explain the delayed scar contracture [6]. Analogous late onset laryngotracheal stenosis has been reported up to 10 years after primary radiotherapy for head and neck cancers [7].

Stoma construction technique is the principal determinant of stenosis risk [8]. Simple circular transection carries the highest incidence (29%–75%), followed by beveled (15%–33%) and flap interposition techniques (0%–8%) [2, 8, 9] (Table 2). Flap interposition disrupts the circular suture line and reduces concentric contracture [1, 5, 8]. Additional risk factors include female sex [1] and infection [1, 3], both identified as independent predictors on multivariate analysis, as well as post-operative radiotherapy [9] (Table 3). In this case, both adjuvant radiotherapy and prior peristomal cellulitis likely contributed.

**Table 2:** Incidence of post-laryngectomy stenosis for various stomal construction techniques

Author, year	Number of cases	Overall stenosis rate (%)	Rate of stenosis for each technique^a^		
			Circular	Beveled	Flap interdigitation
Lam *et al.*, 1983 [2]	141	26	36/116 (31%)	-	1/25 (4%)
Griffith *et al.*, 1982 [9]	89	22	16/56 (29%)	3/20 (15%)	1/13 (8%)
Wax *et al.*, 1995 [8]	106	28	6/8 (75%)	23/70 (33%)	0/28 (0%)

^a^Rate expressed as: Number of cases with stenosis/ number of cases using the technique (%)

**Table 3:** Risk factors and associated incidence of tracheostomal stenosis

Author, Year	n	Radiotherapy					Sex			Tracheostomal infection		
		Pre-operative^a^	Post-operative^a^	Pre and post-operative^a^	None^a^	Statistical significance	Female^a^	Male^a^	Statistical significance	Present^a^	Absent^a^	Statistical significance
Lam *et al.*, 1983 [2]	116	17/57 (30%)			59/116 (32%)	NR	4/9 (44%)	32/107 (30%)	NR			
Griffith *et al.*, 1982 [9]	89	11/57 (19%)	5/14 (36%)	1/6 (17%)	3/12 (25%)	NR						
Kuo *et al.*, 1994 [1]	207	14/128 (11%)	11/66 (17%)	0	2/11 (18%)	NS	6/23 (26%)	21/184 (11%)	*P* < 0.05^b^	4/8 (50%)	28/199 (14%)	*P* < 0.05^b^
Wax *et al.*, 1995 [8]	106	6/29 (21%)	14/42 (33%)	0/2 (0%)	9/28 (32%)	NS	13/28 (46%)	16/74 (22%)	*P* < 0.05^b^	11/33 (33.3%)	NR	NS
de Virgilio *et al.*, 2013 [3]	85									14/24(58%)	15/61 (25%)	*P* < 0.05^b^

^a^Incidence of tracheostomal stenosis, expressed as: cases with risk factor with tracheostomal stenosis/ cases with risk factor (%)

NS: Not significant

NR: Not reported

^b^: Significant in multivariate analysis

Management of tracheostomal stenosis ranges from conservative measures like dilation and stenting, to surgical revision. Conservative approaches often fail to provide durable stomal enlargement [10] and are associated with discomfort and ongoing maintenance. Stomaplasty has been shown to be more effective [5].

Stomaplasty techniques share similar principles with stoma construction: excision of cicatricial tissue, disruption of the circumferential scar and introduction of additional tissue to widen the stomal circumference. Approaches include advancement flaps, V-Y advancements, Z-plasties and star-shaped repairs, all demonstrating favorable outcomes [5, 11, 12] (Table 4). In patients with an existing or planned tracheoesophageal puncture, posterior stomal incisions are avoided. Techniques described by Campbell and Kim employ lateral or anterior flap interposition to preserve the posterior tracheal wall [10, 13]. Seo’s described a technique that addresses both tracheal and stomal stenosis, while preserving a tracheoesophageal puncture. This technique involved segmental tracheal resection, preservation of the posterior wall of mucosa surrounding the tracheoesophageal puncture, and inset of a skin flap into the anterior tracheal wall [4].

**Table 4:** Stomaplasty techniques and success rates

Author, year	n	Prior radiotherapy^a^	Stomaplasty technique	Success (Tube not required)^b^	Partial success (Tube required intermittently)^b^	Failure (Tube dependent)^b^	Follow up period
Campbell *et al.*,1997 [10]	15	14/15 (93%)	Tracheal advancement, anterior skin flap interdigitation	7/15 (47%)	6/15 (40%)	2/15 (13%)	6 to 48 months
Bretteville *et al.*, 1992 [11]	20	19/20 (95%)	Radial skin incisions, multiple Y-V plasties	19/20 (95%) of patients had an increase in stoma size post stomaplasty (on average by a factor of 3.8). Tube requirement not documented.			Minimum 2 years
Kim et. al, 2010 [13]	5	3/5 (60%)	Double reversing Z-plasty with inferiorly widening stomaplasty	5/5 (100%)	0/5 (0%)	0/5 (0%)	8 months to 5 years
Wax *et al.*,1999 [5]	9	NR	Advancement flaps	8/9 (89%)		1/9 (11%)	NR
	15		Z-plasty	13/15 (87%)		2/15 (13%)	
	8		V^-^Y inset	8/8 (100%)		0/8 (0%)	
	6		Other procedures	3/6 (50%)		3/6 (50%)	
	7		Dilation	2/7 (29%)		5/7 (71%)	

^a^Patients with prior radiotherapy, expressed as: number of patients with prior radiotherapy/ n (%)

^b^Rates expressed as: Number of cases with success, partial success or failure / Number of cases using the technique (%)

NR: Not reported

Our technique incorporates prior established principles in a straightforward manner. Scar excision addressed the tracheocutaneous contracture, while lateral tracheal incisions with advancement flap interposition introduced additional tissue and disrupted circular contracture forces. The approach avoids a circumferential incision and extensive paratracheal dissection, potentially reducing wound complications in irradiated tissue. Importantly, the posterior tracheal wall is preserved, maintaining the option for tracheoesophageal voice rehabilitation.

## Conclusion

Although limited by the single-case nature and short follow up, this report highlights the potential for delayed tracheostomal stenosis after laryngectomy and demonstrates a simple, effective technique for its management.

## References

[1] Kuo M, Ho CM, Wei WI et al. Tracheostomal stenosis after total laryngectomy: an analysis of predisposing clinical factors. Laryngoscope 1994;104:59–63. 10.1288/00005537-199401000-00010 8295457

[2] Lam KH, Wei WI, Wong J et al. Tracheostome construction during laryngectomy--a method to prevent stenosis. Laryngoscope 1983;93:212–5. 10.1288/00005537-198302000-00018 6337312

[3] de Virgilio A, Greco A, Gallo A et al. Tracheostomal stenosis clinical risk factors in patients who have undergone total laryngectomy and adjuvant radiotherapy. Eur Arch Otorhinolaryngol 2013;270:3187–9. 10.1007/s00405-013-2695-6 24057098

[4] Seo GT, Wein LE, Dowling EM et al. A novel technique for management of stenosis of the postlaryngectomy stoma with preservation of a functional tracheoesophageal puncture following tracheal resection. Head Neck 2022;44:1737–41. 10.1002/hed.27048 35388943

[5] Wax MK, Touma BJ, Ramadan HH. Management of tracheostomal stenosis. Laryngoscope 1999;109:1397–401. 10.1097/00005537-199909000-00006 10499042

[6] Queenan N, Trivedi J, Bertoni D et al. Characterizing radiation-related laryngotracheal stenosis. Am J Otolaryngol 2025;46:104643. 10.1016/j.amjoto.2025.104643 40311495

[7] Stevens MS, Chang A, Simpson CB. Supraglottic stenosis: etiology and treatment of a rare condition. Ann Otol Rhinol Laryngol 2013;122:205–9. 10.1177/000348941312200310 23577574

[8] Wax MK, Touma BJ, Ramadan HH. Tracheostomal stenosis after laryngectomy: incidence and predisposing factors. Otolaryngol Head Neck Surg 1995;113:242–7. 10.1016/S0194-5998(95)70112-5 7675484

[9] Griffith GR, Luce EA. Tracheal stomal stenosis after laryngectomy. Plast Reconstr Surg 1982;70:694–8. 10.1097/00006534-198212000-00006 7146151

[10] Campbell BH, Rubach BW, McAuliffe TL et al. Tracheal advancement flap for postlaryngectomy stomal stenosis. Head Neck 1997;19:211–5. 10.1002/(SICI)1097-0347(199705)19:3<211::AID-HED8>3.0.CO;2-5 9142521

[11] Bretteville G, Söberg R, Boysen M. An improved technique for treating tracheostomal stenosis following laryngectomy. Clin Otolaryngol Allied Sci 1992;17:44–8. 10.1111/j.1365-2273.1992.tb00986.x 1555317

[12] Giacomarra V, Russolo M, Tirelli G et al. Surgical treatment of tracheostomal stenosis. Laryngoscope 2001;111:1281–4. 10.1097/00005537-200107000-00026 11568555

[13] Kim YH, Kim NH, Seong SY et al. Double reversing Z-plasty with inferiorly widening stomaplasty for the management of tracheostomal stenosis. Auris Nasus Larynx 2010;37:361–4. 10.1016/j.anl.2009.10.002 20042304

